# Differences in Clinical Characteristics and Chest Images between Coronavirus Disease 2019 and Influenza-Associated Pneumonia

**DOI:** 10.3390/diagnostics11020261

**Published:** 2021-02-08

**Authors:** Si-Ho Kim, Yu Mi Wi, Sujin Lim, Kil-Tae Han, In-Gyu Bae

**Affiliations:** 1 Division of Infectious Diseases, Samsung Changwon Hospital, Sungkyunkwan University School of Medicine, Changwon 51353, Korea; wychhazel@naver.com; 2 Department of Pulmonology Diseases, Gyeongsangnam-do Masan Medical Center, Changwon 51264, Korea; tnwls0311@naver.com; 3 Department of Radiology, Gyeongsangnam-do Masan Medical Center, Changwon 51264, Korea; jinijjim@hanmail.net; 4 Division of Infectious Diseases, Gyeongsang National University Hospital, Gyeongsang National University School of Medicine, Jinju 52727, Korea; ttezebae@gmail.com

**Keywords:** severe acute respiratory syndrome coronavirus 2, COVID-19, influenza, human, X-rays, tomography, X-ray computed

## Abstract

Background: Concerns are arising about the simultaneous occurrence of the coronavirus disease 2019 (COVID-19) pandemic and the influenza epidemic, the so-called “twindemic”. In this study, we compared clinical characteristics and chest images from patients with COVID-19 and influenza. Methods: We conducted a case-control study of COVID-19 and age- and sex-matched influenza patients. Clinical characteristics and chest imaging findings between patients with COVID-19 and matched influenza patient controls were compared. Results: A total of 47 patients were enrolled in each group. Anosmia (14.9%) and ageusia (21.3%) were only observed in COVID-19 patients. There were 31 (66%) and 23 (48.9%) patients with COVID-19 and influenza who had pulmonary lesions confirmed by chest computed tomography (CT), respectively. The interval between symptom onset and pneumonia was significantly longer in patients with COVID-19. Round opacities were more common in images from COVID-19 patients (41.9% vs. 8.7%, *p* = 0.007), whereas pure consolidation (0% vs. 34.9%, *p* < 0.001) and pleural effusion (0% vs. 17.4%, *p* = 0.028) were more common in images from influenza patients. Notably, the difference in the number of involved pulmonary lobes observed on CT and pulmonary fields observed on radiographic images was significantly higher in COVID-19-associated pneumonia than that in influenza-associated pneumonia (2.32 ± 1.14 vs. 1.48 ± 0.99, *p* = 0.010). Conclusions: Chest images and thorough review of clinical findings could provide value for proper differential diagnoses of COVID-19 patients, but they are not sufficiently sensitive for initial diagnoses. In addition, chest radiography could underestimate COVID-19 lung involvement because of the lesion characteristics of COVID-19-associated pneumonia.

## 1. Introduction

Since the first case was reported in Wuhan, China, in December 2019, coronavirus disease 2019 (COVID-19), caused by severe acute respiratory syndrome coronavirus 2 (SARS-CoV-2), has rapidly spread worldwide, with more than 39 million confirmed cases on 17 October 2020 [1,2]. Recent studies have described the distinctive clinical characteristics and images of COVID-19 compared to other respiratory viral diseases, and chest computed tomography (CT) could help the diagnosis and outcome prediction of patients with COVID-19 [3,4,5]. Although a diagnosis cannot be achieved on the basis of imaging features alone, identifying specific viral pneumonia patterns may enable differentiation between viral pathogens [6]. In addition, concerns are arising about the simultaneous occurrence of the COVID-19 pandemic and the influenza epidemic, the so-called “twindemic”, as the COVID-19 pandemic is continuing more aggressively [7]. Therefore, a proper differential diagnosis of the two diseases can be an important factor in improving a patient’s prognosis and maintaining a national health system.

In this study, we described and compared the clinical characteristics and chest images from patients with COVID-19-associated pneumonia and influenza-associated pneumonia to evaluate their clinical usefulness.

## 2. Patients and Methods

### 2.1. Study Population

We conducted a case-control study of COVID-19 and age- and sex-matched influenza patients. Only adult patients (≥18 year-old) were included. Case patients with COVID-19 who had undergone chest CT were retrospectively enrolled from the Masan Medical Center (MMC) between 25 February 2020 and 1 April 2020. Since February 2020, the MMC has been a dedicated national center in Korea for the management of confirmed COVID-19 patients with mild-to-moderate disease severity. Control patients with influenza who had undergone chest CT were retrospectively enrolled from the Samsung Changwon Hospital, a secondary care academic hospital, from January 2016 to March 2020. A confirmed case of SARS-CoV-2 infection was diagnosed by COVID-19 case definitions from the World Health Organization [2]. In this study, SARS-CoV-2 infections were confirmed with RT-PCR, and influenza was diagnosed with a rapid influenza diagnostic test or a multiplex PCR test for the respiratory virus. Additional fever studies were decided and prescribed by the attending physician for each patient. The usual practice for fever and/or pneumonia study included two sets of blood culture and sputum culture. Patients without chest CT and those with interstitial lung disease were excluded from our study.

### 2.2. Chest Images and Clinical Data Acquisition

A baseline digital posteroanterior or anteroposterior chest radiography was taken for all of the study participants at full inspiration using a chest radiograph machine (Innovision-SH, DK, Seoul, Korea; Sirium 130HP mobile X-ray machine, Hitachi, Ltd., Chiyoda-ku, Tokyo, Japan).

Patients with abnormal findings on chest radiography or desaturation (SpO_2_ < 95% measured by pulse oximetry) were indicated for chest CT. Chest CT was conducted using a 16-slice Philips Brilliance multi-slice CT scanner with the following parameters: tube voltage of 120 kV, tube current-exposure time product of 150 mAs, scan thickness of 5 mm, and reconstruction thickness of 5 mm. Chest CT sections were obtained. Images were obtained and compared with both mediastinal (width, 350–450 HU; level, 20 to 40 HU) and parenchymal (width, 1200–1600 HU; level, −500 to 700 HU) window setting. Clinical data and laboratory data of patients were collected from electronic medical records. Because of data incompleteness, only patients with pneumonia having a laboratory test in each group were compared. The severity of diseases was evaluated by the National Early Warning Score (NEWS) [8].

### 2.3. Image Interpretation

An attending radiologist and a physician evaluated the chest radiographs and CT images by consensus. In the chest CT evaluation, the involved pulmonary lobe and the shape, density, and the axial location of the lung lesion were recorded. If a lesion was located in the outer one-third of the lung, it was classified as peripherally located. If the lesion was located in the inner two-thirds of the lung, it was classified as centrally located, consistent with a previous study [9]. Lung involvement on chest radiographs was recorded according to the following five fields: right upper, right middle, right lower, left upper/middle, and left lower lung.

### 2.4. Statistical Analysis

All statistical analyses were performed using SPSS 23.0 for Window (IBM Corp., 2015, Chicago, IL, USA). To compare characteristics between COVID-19-associated pneumonia and influenza-associated pneumonia groups, Student’s *t*-test or Mann–Whitney test was used to compare continuous variables of two groups, and one-way ANOVA or Kruskal–Wallis test was used to compare continuous variables of multiple groups. Categorical variables were compared by using the chi-square test or Fisher’s exact test. All *p* values were two-tailed, and *p* values < 0.05 were considered statistically significant.

## 3. Results

### 3.1. Study Population and Clinical Findings

A total of 47 patients were enrolled in both the COVID-19 group and the influenza group. No co-infection was documented in the COVID-19 group, however, respiratory syncytial virus (one case) and *Haemophilus influenzae* (one case) were suspected co-infections in the influenza group. The female to male ratio in each cohort was 3:2. Ages were similar in both groups (49.04 ± 13.87 and 49.23 ± 14.33 years in the COVID-19 group and the influenza group, respectively). The timing of symptoms onset in patients of the COVID-19 and influenza group before conducting chest CT ranged from −3 to 29 days and 1 to 15 days, respectively. NEWS was higher in the influenza group (median 3, interquartile range (IQR) 2–5) than the COVID-19 group (median 1, IQR 0–2) (*p* < 0.001).

The most common symptoms were fever/chill (66.0% vs. 80.9%, *p* = 0.102) and cough (36.2% and 46.8%, *p* = 0.403) in both groups. Anosmia (14.9% vs. 0 %, *p* = 0.012) and ageusia (21.3% vs. 0%, *p* = 0.001) were only presented in patients with COVID-19 infection (Table 1).

### 3.2. Chest Images and Laboratory Findings

Among the patients with COVID-19 and influenza, only 31 (66%) and 23 (48.9%) patients, respectively, had pulmonary lesions confirmed by chest CT. The interval between symptom onset and CT scan was significantly longer in patients with COVID-19-associated pneumonia (median 10 days vs. 2 days, *p* > 0.001).

Among patients with pneumonia, 12 with COVID-19 (12/31, 38.7%) and 9 (9/23, 39.1%) with influenza-associated pneumonia were not evaluated by chest radiography. No differences were observed between COVID-19-associated pneumonia and influenza-associated pneumonia patients in the number of involved pulmonary lobes (3.39 ± 1.61 vs. 3.17 ± 1.37, *p* = 0.530) in chest CT or pulmonary fields (1.06 ± 1.15 vs. 1.70 ± 1.66, *p* = 0.182) in chest radiography. The right and left lower lobes were most frequently involved in both groups. Both COVID-19-associated pneumonia and influenza-associated pneumonia mostly presented as pure glass-ground opacity (GGO) on chest CT (58.1% vs. 39.1%, *p* = 0.169); however, no pneumonia with pure consolidation was observed in COVID-19 images (0% vs. 34.9%; *p* < 0.001). Round opacities were more frequently observed in COVID-19-associated pneumonia (41.9% vs. 8.7%; *p* = 0.007), and pleural effusion was only seen in influenza-associated pneumonia (0% vs. 17.4%, *p* = 0.028). Pulmonary lesions tended to be located more peripherally in COVID-19 than in influenza (61.3% vs. 43.5%; *p* = 0.194). Notably, the difference in the number of involved pulmonary lobes observed on CT and pulmonary fields observed on radiographic images was significantly higher in COVID-19-associated pneumonia than that in influenza-associated pneumonia (2.32 ± 1.14 vs. 1.48 ± 0.99, *p* = 0.010) (Table 2).

Table 3 describes the results of patients having laboratory tests between COVID-19-associated pneumonia and influenza-associated pneumonia. Whereas leukocytosis (0% vs. 40.9%) was more frequently observed in influenza-associated pneumonia, leukopenia was more common in COVID-19-associated pneumonia (18.5% vs. 4.5%) (*p* < 0.001). Patients with influenza-associated pneumonia showed more elevated C-reactive protein (CRP) than those with COVID-19-associated pneumonia (44.4% vs. 90.9%, *p* = 0.001).

## 4. Discussion

Our study demonstrates that significant differences exist between features identified in CT images of COVID-19-associated pneumonia and influenza-associated pneumonia. Additionally, we documented meaningful discordance between chest radiographs and CT scans in patients with COVID-19-associated pneumonia. Our findings show that chest radiographs did not reflect the actual status of patients with COVID-19-associated pneumonia. In addition, unique symptoms of COVID-19, such as anosmia, ageusia, and later onset of pneumonia, could differentiate COVID-19 patients from influenza patients.

Typical and frequently observed CT imaging findings from patients with COVID-19 have been previously described, and include GGO, bilateral lesions, peripheral distribution, and multi-lobular involvement [3,9,10]. The most common characteristic in CT images from patients with influenza-associated pneumonia was GGO, which is similar to COVID-19; however, unlike COVID-19, GGO in influenza-associated pneumonia is distributed relatively uniformly over both peripheral and central areas. In addition, confluent consolidation and pleural effusion have been frequently reported in influenza-associated pneumonia [6,11]. Meanwhile, several Chinese studies have directly compared CT findings of COVID-19-associated pneumonia with influenza associated-pneumonia [12,13,14]. For example, Liu et al. reported that COVID-19-associated pneumonia had more rounded opacities (35% vs. 17%) and interlobular septal thickening (66% vs. 43%). In contrast, influenza-associated pneumonia had more pleural effusion (6% vs. 31%) [12]. Another study indicated that pulmonary lesions of COVID-19-associated pneumonia were located more peripherally than that of influenza [13]. In accordance with the previous studies, we found that rounded opacity was more frequently observed in COVID-19-associated pneumonia, whilst consolidative lesion and pleural effusion were more frequently observed in influenza-associated pneumonia. Interestingly, COVID-19-associated pneumonia showed a similar number of involved pulmonary lobes even with less severe symptoms than those with influenza-associated pneumonia. In addition, only 61.3% (19/31) of chest radiographs could detect COVID-19-associated pneumonia confirmed by chest CT. These findings related to chest image could explain our novel finding of the study that COVID-19 lung involvement was highly underestimated in chest radiograph analyses compared to chest CT. These outcomes could possibly be related to the characteristics of COVID-19 pulmonary lesions, such as GGO, peripheral location, and the absence of pleural effusion.

An early study from China suggested that chest CT sensitivity was 97% based on positive COVID-19 RT-PCR results [15]. In the study, patients were all from the central area of the outbreak of COVID-19 in Wuhan, China. However, our study demonstrated that chest CT could provide a diagnosis in only 66% of patients with COVID-19. This finding is consistent with that of a previous study [10], in which only 44% of patients with early COVID-19 infection showed pulmonary lesions in chest CT images. Korean response system using extensive viral screening of COVID-19 and rapid contact tracing resulted in the early detection of confirmed patients [16]. Because viral shedding of COVID-19 begins early or even before symptoms appear [17], CT scan diagnoses might be less sensitive than the current RT-PCR method in the patients who were in the early stages. In addition, our study showed that patients with COVID-19-associated pneumonia had a longer interval from symptoms onset to pneumonia. A previous study reported that clinical stages of COVID-19 disease could be classified by early (stage 1), pulmonary (stage 2), and hyper-inflammation phase (stage 3) [18]. Pneumonia usually occurs in stage 2, which corresponds to 7–8 days from the onset of symptoms [16,18,19]. In contrast, prior data suggest influenza-associated pneumonia usually occurs 2–5 days after typical influenza symptom onset [20,21]. One French study emphasized that clinical aggravation was later in patients with COVID-19 than influenza [22]. Therefore, these findings suggest that COVID-19-associated pneumonia might develop later from the onset of symptoms than influenza pneumonia. The American College of Radiology recommends the use of chest radiographs and CT scans for suspected COVID-19 infection only in limited circumstances [23]. Therefore, chest CT scans may be most helpful for providing information to prioritize pneumonia patients for COVID-19 tests, rather than for early diagnoses, particularly when resources are limited. Although our study did not evaluate the diagnostic performance of lung ultrasound, lung ultrasounds have been previously studied for diagnosing and evaluating the clinical course of patients with COVID-19. A prior study reported that point-of-care lung ultrasound had a sensitivity of 89% and specificity of 59% [24]. Even though there is no known pathognomic ultrasound finding for COVID-19, ultrasound can detect pulmonary dynamics for patients with COVID-19 [25]. Therefore, lung ultrasound should be considered an important image modality for diagnosing and managing COVID-19 infection without exposure to radiation.

In addition to the difference in radiologic findings, clinical differences between COVID-19 and influenza were observed in this study. Anosmia and ageusia were significantly different symptoms between each group. Among laboratory findings, leukocytosis and leukopenia were predominant in patients with influenza-associated and COVID-19-associated pneumonia, respectively. This was in line with prior studies that reported frequently observed leukopenia and anosmia in patients with COVID-19 infection [22,26]. These findings suggest that a thorough review of the clinical presentations and laboratory findings of each patient could give diagnostic clues for differentiating COVID-19 and influenza among patients with respiratory symptoms.

Our study had several limitations. First, the disease severity of COVID-19 appeared lower than that of influenza in the controls. The low-severity of COVID-19 could be related to the discordance between chest radiographs and CT images. For example, consolidation is dominantly observed in severe and late-stage COVID-19-associated pneumonia. [3] Because only mild-to-moderate COVID-19 patients were managed in our medical center, no pure consolidation was observed in the chest CT of patients with COVOD-19-associated pneumonia. Frequently observed CRP elevation in influenza-associated pneumonia seemed to be associated with disease severity. Consequently, our findings could not be generalized to patients with severe pneumonia. However, our study showed an interesting finding in that numerically more pneumonia was observed in COVID-19 patients with lesser severity compared to influenza patients. This might reflect the disease characteristics of COVID-19 compared to influenza. Second, there was a difference in the timing of symptoms onset before conducting CT in COVID-19 and influenza patients. This could be a bias for a matched case-control study. However, our study and other prior studies have shown that the onset of pneumonia and disease aggravation was different between patients with COVID-19-associated pneumonia and influenza-associated pneumonia. This suggests that matching the timing of CT evaluation might not be appropriate for our study. Third, the genotype of SARS-CoV-2 and influenza was not evaluated. The possibility of differences in the characteristics of chest images according to different genotypes cannot be excluded.

Finally, due to the limitation of retrospective study design, the probability of co-infection caused by atypical bacteria was not fully investigated. However, the characteristics of our study populations were in line with prior studies, and this limitation might not distort real clinical circumstances.

In conclusion, chest images and thorough review of clinical presentations could provide value for proper differential diagnoses between COVID-19-associated pneumonia and influenza-associated pneumonia and help improve clinical outcomes of patients with a respiratory viral illness in preparation for the “twindemic”. However, they are not sufficiently sensitive for initial diagnoses of patients with suspected COVID-19 infection. Notably, chest radiography could underestimate COVID-19 lung involvement. Therefore, physicians should be aware of the appropriate diagnostic value of chest images for COVID-19 and interpret them in the context of clinical symptoms, signs, and other laboratory data.

## Figures and Tables

**Table 1:** Comparison between the clinical characteristics of patients with coronavirus disease 2019 (COVID-19) and influenza virus infection.

	COVID-19 (47)	Influenza (47)	*p*-Value
**Patient characteristics**			
Age (mean ± standard deviation)	49.04 ± 13.87	49.23 ± 14.33	0.948
Female	28 (59.6)	28 (59.6)	>0.999
Arterial hypertension	8 (17.0)	6 (12.8)	0.562
Diabetes mellitus	6 (12.8)	5 (10.6)	0.748
Heart failure with reduced ejection fraction	1 (2.1)	2 (4.3)	>0.999
Bronchial asthma	0 (0.0)	1(2.1)	>0.999
Liver cirrhosis	1 (2.1)	1 (2.1)	>0.999
Malignancy ^a^	1 (2.1)	1 (2.1)	>0.999
Symptoms onset (median days before CT evaluation, interquartile range)	8 (2.75–17)	2 (1–4.5)	>0.001
NEWS score, median (interquartile range)	1 (0–2)	3 (2–5)	>0.001
**Symptoms**			
Dyspnea **^b^**	5 (10.6)	11 (23.4)	0.100
Fever/chill	31 (66.0)	38 (80.9)	0.102
Cough	17 (36.2)	22 (46.8)	0.403
Headache	5 (10.6)	5 (10.6)	>0.999
Nasal congestion/rhinorreha	4 (8.5)	6 (12.8)	0.740
Sore throat	9 (19.1)	6 (12.8)	0.398
Chest pain	2 (4.3)	4 (8.5)	0.677
Gastrointestinal symptoms	0 (0.0)	5 (10.6)	0.056
Anosmia	7 (14.9)	0 (0.0)	0.012
Ageusia	10 (21.3)	0 (0.0)	0.001

NOTE: Data represent the number (%) of patients unless otherwise specified. ^a^ 1 patient with colon cancer (COVID-19 group) and 1 patient with multiple myeloma (influenza group). ^b^ Dyspnea was reported by the patient or characterized as ≥class II of the New York Heart Association Functional Classification; CT: computed tomography; NEWS: National Early Warning Score.

**Table 2:** Comparison between clinical characteristics and chest imaging findings of patients having pulmonary lesions with coronavirus disease (COVID-19)-associated pneumonia and influenza-associated pneumonia.

	COVID-19 (31)	Influenza (23)	*p*-Value
**Patient characteristics**			
Age (mean ± standard deviation)	49.97 ± 14.41	54.00 ± 11.72	0.217
Female	20 (64.5)	15 (65.2)	0.957
Arterial hypertension	4 (12.9)	5 (21.7)	0.472
Diabetes mellitus	5 (16.1)	2 (8.7)	0.685
Heart failure with reduced ejection fraction	1 (3.2)	1 (4.3)	>0.999
Bronchial asthma	0 (0.0)	0 (0.0)	N/A
Liver cirrhosis	1 (3.2)	1 (4.3)	>0.999
Malignancy ^a^	1 (3.2)	1 (4.3)	>0.999
Symptoms onset (median days before CT evaluation, interquartile range)	10 (3–17)	2 (1.5–5.5)	>0.001
NEWS score, median (interquartile range)	1 (0–2)	4 (2–6)	>0.001
**CT results: involved pulmonary lobe**			
Right upper lobe	18 (58.1)	13 (56.5)	0.910
Right middle lobe	15 (48.4)	12 (52.2)	0.783
Right lower lobe	26 (83.9)	18 (78.3)	0.728
Left upper lobe	20 (64.5)	9 (39.1)	0.064
Left lower lobe	26 (83.9)	21 (91.3)	0.685
The number of involved lobes in chest CT, mean ± standard deviation and median (interquartile range)	3.39 ± 1.61 4 (2–5)	3.17 ± 1.37 3 (2–4)	0.530
**CT results: characteristics of pulmonary lesions**			
Pure ground-glass opacity	18 (58.1)	9 (39.1)	0.169
Pure consolidation	0 (0.0)	8 (34.9)	>0.001
Mixed ground-glass opacity and consolidation	13 (41.9)	6 (26.1)	0.228
Round opacity	13 (41.9)	2 (8.7)	0.007
Pleural effusion not associated with heart failure	0 (0.0)	4 (17.4)	0.028
Lymphadenopathy	5 (16.1)	9 (39.1)	0.056
**CT results: axial location of pulmonary lesions**			
Peripherally located only	19 (61.3)	10 (43.5)	0.194
Centrally located only	3 (9.7)	4 (17.4)	0.443
Peripherally and centrally located	9 (29.0)	9 (38.1)	0.436
**Chest radiography results: involved pulmonary lobe**			
Right upper lung field	2 (6.5)	5 (21.7)	0.122
Right middle lung field	2 (6.5)	6 (26.1)	0.060
Right lower lung field	12 (38.7)	13 (56.5)	0.194
Left upper lung field	1 (3.2)	3 (13.0)	0.301
Left middle and lower lung field	16 (51.6)	12 (52.2)	0.967
The number of involved lung fields on chest x-ray, mean ± standard deviation, and median (interquartile range)	1.06 ± 1.15 1 (0–2)	1.70 ± 1.66 2 (0–3)	0.182
**Pneumonia detected only by CT, not by chest radiography** no. (%)	12 (38.7)	9 (39.1)	0.975
**Difference in the number of involved pulmonary lobes in chest CT and pulmonary fields in radiography**, mean ± standard deviation and median (interquartile range)	2.32 ± 1.14 2 (1–3)	1.48 ± 0.99 1 (1–2)	0.010

NOTE: Data represent the number (%) of patients unless otherwise specified. ^a^ 1 patient with colon cancer (COVID-19 group) and 1 patient with multiple myeloma (influenza group); CT: computed tomography; NEWS: National Early Warning Score.

**Table 3:** Comparison between clinical characteristics and chest imaging findings of patients having pulmonary lesions with coronavirus disease (COVID-19)-associated pneumonia and influenza-associated pneumonia.

	COVID-19 (27)	Influenza (22)	*p*-Value
Leukocyote			<0.001
Leukocytosis (>11,000/mm^3^)	0 (0.0)	9 (40.9)	
Normal range	22 (81.5)	12 (54.5)	
Leukopenia (<4000/mm^3^)	5 (18.5)	1 (4.5)	
Thrombocytopenia (<140,000/mm^3^)	3 (11.1)	3 (13.6)	>0.999
Elevated C-reactive protein (>0.5 mg/dL)	12 (44.4)	20 (90.9)	0.001
Hypoalbuminemia (<3.0 g/dL)	0 (0.0)	1 (4.5)	0.449
Elevated transaminase *	7 (25.9)	8 (36.4)	0.367

* Cut-off value: >40 U/L for male, >32 U/L for female.

## References

[1] Zhu N., Zhang D., Wang W., Li X., Yang B., Song J., Zhao X., Huang B., Shi W., Lu R. (2020). A Novel Coronavirus from Patients with Pneumonia in China, 2019. N. Engl. J. Med..

[2] WHO Official Updates-Coronavirus Disease 2019. https://www.who.int/emergencies/diseases/novel-coronavirus-2019.

[3] Salehi S., Abedi A., Balakrishnan S., Gholamrezanezhad A. (2020). Coronavirus Disease 2019 (COVID-19): A Systematic Review of Imaging Findings in 919 Patients. Am. J. Roentgenol..

[4] Feng Z., Yu Q., Yao S., Luo L., Duan J., Yan Z., Yang M., Tan H., Ma M., Li T. (2020). Early Prediction of Disease Progression in 2019 Novel Coronavirus Pneumonia Patients Outside Wuhan with CT and Clinical Characteristics. medRxiv.

[5] Bai H.X., Hsieh B., Xiong Z., Halsey K., Choi J.W., Tran T.M.L., Pan I., Shi L.-B., Wang D.-C., Mei J. (2020). Performance of Radiologists in Differentiating COVID-19 from Non-COVID-19 Viral Pneumonia at Chest CT. Radiology.

[6] Kim M.Y., Lim S., Choe J., Choi S.H., Sung H., Do K.-H. (2018). Radiographic and CT Features of Viral Pneumonia. Radiographics.

[7] Jaklevic M.C. (2020). Flu Vaccination Urged During COVID-19 Pandemic. JAMA.

[8] Smith G.B., Prytherch D.R., Meredith P., Schmidt P.E., Featherstone P.I. (2013). The ability of the National Early Warning Score (NEWS) to discriminate patients at risk of early cardiac arrest, unanticipated intensive care unit admission, and death. Resuscitation.

[9] Yoon S.H., Lee K.H., Kim J.Y., Lee Y.K., Ko H., Kim K.H., Park C.M., Kim Y.-H. (2020). Chest Radiographic and CT Findings of the 2019 Novel Coronavirus Disease (COVID-19): Analysis of Nine Patients Treated in Korea. Korean J. Radiol..

[10] Bernheim A., Mei X., Huang M., Yang Y., Fayad Z.A., Zhang N., Diao K., Lin B., Zhu X., Li K. (2020). Chest CT Findings in Coronavirus Disease-19 (COVID-19): Relationship to Duration of Infection. Radiology.

[11] Aviram G., Bar-Shai A., Sosna J., Rogowski O., Rosen G., Weinstein I., Steinvil A., Zimmerman O. (2010). H1N1 Influenza: Initial Chest Radiographic Findings in Helping Predict Patient Outcome. Radiology.

[12] Liu M., Zeng W., Wen Y., Zheng Y., Lv F., Xiao K. (2020). COVID-19 pneumonia: CT findings of 122 patients and differentiation from influenza pneumonia. Eur. Radiol..

[13] Lin L., Fu G., Chen S., Tao J., Qian A., Yang Y., Wang M. (2020). CT Manifestations of Coronavirus Disease (COVID-19) Pneu-monia and Influenza Virus Pneumonia: A Comparative Study. AJR Am. J. Roentgenol..

[14] Yin Z., Kang Z., Yang D., Ding S., Luo H., Xiao E. (2020). A Comparison of Clinical and Chest CT Findings in Patients with In-fluenza A (H1N1) Virus Infection and Coronavirus Disease (COVID-19). AJR Am. J. Roentgenol..

[15] Ai T., Yang Z., Hou H., Zhan C., Chen C., Lv W., Tao Q., Sun Z., Xia L. (2020). Correlation of Chest CT and RT-PCR Testing for Coronavirus Disease 2019 (COVID-19) in China: A Report of 1014 Cases. Radiology.

[16] Wi Y.M., Lim S.J., Kim S.-H., Lim S., Lee S.J., Ryu B.-H., Hong S.I., Cho O.-H., Moon K., Hong K.-W. (2020). Response System for and Epidemiological Features of COVID-19 in Gyeongsangnam-do Province in South Korea. Clin. Infect. Dis..

[17] Wölfel R., Corman V.M., Guggemos W., Seilmaier M., Zange S., Müller M.A., Niemeyer D., Jones T.C., Vollmar P., Rothe C. (2020). Virological assessment of hospitalized patients with COVID-2019. Nat. Cell Biol..

[18] Siddiqi H.K., Mehra M.R. (2020). COVID-19 illness in native and immunosuppressed states: A clinical–therapeutic staging proposal. J. Hear. Lung Transplant..

[19] Suh H.J., Kim D.H., Heo E.Y., Lee H.W., Lee J.-K., Lee C.-S., Kim M., Jeon Y.D., Chung J.-W., Kim Y.K. (2020). Clinical Characteristics of COVID-19: Clinical Dynamics of Mild Severe Acute Respiratory Syndrome Coronavirus 2 Infection Detected by Early Active Surveillance. J. Korean Med. Sci..

[20] Wi Y.M., Kim J.M., Peck K.R. (2013). Serum albumin level as a predictor of intensive respiratory or vasopressor support in influenza A (H1N1) virus infection. Int. J. Clin. Pract..

[21] Rello J., Pop-Vicas A. (2009). Clinical review: Primary influenza viral pneumonia. Crit. Care.

[22] Zayet S., Kadiane-Oussou N.J., Lepiller Q., Zahra H., Royer P.-Y., Toko L., Gendrin V., Klopfenstein T. (2020). Clinical features of COVID-19 and influenza: A comparative study on Nord Franche-Comte cluster. Microbes Infect..

[23] American College of Radiology (2020). ACR Recommendations for the Use of Chest Radiography and Computed Tomography (CT) for Suspected COVID-19 Infection.

[24] Haak S.L., Renken I.J., Jager L.C., Lameijer H., Van Der Kolk B. (2020). (Britt) Y. Diagnostic accuracy of point-of-care lung ultrasound in COVID-19. Emerg. Med. J..

[25] Allinovi M., Parise A., Giacalone M., Amerio A., Delsante M., Odone A., Franci A., Gigliotti F., Amadasi S., Delmonte D. (2020). Lung Ultrasound May Support Diagnosis and Monitoring of COVID-19 Pneumonia. Ultrasound Med. Biol..

[26] Qu J., Chang L.K., Tang X., Du Y., Yang X., Liu X., Han P., Xue Y. (2020). Clinical characteristics of COVID-19 and its comparison with influenza pneumonia. Acta Clin. Belg..

